# Human peritoneal fluid exerts ovulation- and nonovulation-sourced oncogenic activities on transforming fallopian tube epithelial cells

**DOI:** 10.1186/s12935-024-03406-1

**Published:** 2024-07-02

**Authors:** Che-Fang Hsu, Vaishnavi Seenan, Liang-Yuan Wang, Pao-Chu Chen, Dah-Ching Ding, Tang-Yuan Chu

**Affiliations:** 1 Center for Prevention and Therapy of Gynecological Cancers, Department of Medical Research, Hualien Tzu Chi Hospital, Buddhist Tzu Chi Medical Foundation, Hualien, 970 Taiwan; 2 Institute of Medical Sciences, Tzu Chi University, Hualien, 970 Taiwan; 3 Department of Molecular Biology and Human Genetics, Tzu Chi University, Hualien, 970 Taiwan; 4 Department of Obstetrics & Gynecology, Hualien Tzu Chi Hospital, Buddhist Tzu Chi Medical Foundation, 707, Section 3, Chung-Yang Road, Hualien, 970 Taiwan

**Keywords:** High-grade serous carcinoma, Fallopian tube epithelium, Follicular fluid, Peritoneal fluid, Peritoneal seeding

## Abstract

Secretory cells in the fallopian tube fimbria epithelium (FTE) are regarded as the main cells of origin of ovarian high-grade serous carcinoma (HGSC). Ovulation is the main cause of FTE oncogenesis, which proceeds through a sequence of TP53 mutations, chromosomal instability due to Rb/cyclin E aberration, in situ carcinoma (STIC), and metastasis to the ovary and peritoneum (metastatic HGSC). Previously, we have identified multiple oncogenic activities of the ovulatory follicular fluid (FF), which exerts the full spectrum of transforming activity on FTE cells at different stages of transformation. After ovulation, the FF is transfused into the peritoneal fluid (PF), in which the FTE constantly bathes. We wondered whether PF exerts the same spectrum of oncogenic activities as done by FF and whether these activities are derived from FF. By using a panel of FTE cell lines with p53 mutation (FT282-V), p53/CCNE1 aberrations (FT282-CCNE1), and p53/Rb aberrations plus spontaneous transformation, and peritoneal metastasis (FEXT2), we analyzed the changes of different transformation phenotypes after treating with FF and PF collected before or after ovulation. Similar to effects exhibited by FF, we found that, to a lesser extent, PF promoted anchorage-independent growth (AIG), migration, anoikis resistance, and peritoneal attachment in transforming FTE cells. The more transformed cells were typically more affected. Among the transforming activities exhibited by PF treatment, AIG, Matrigel invasion, and peritoneal attachment growth were higher with luteal-phase PF treatment than with the proliferative-phase PF treatment, suggesting an ovulation source. In contrast, changes in anoikis resistance and migration activities were similar in response to treatment with PF collected before and after ovulation, suggesting an ovulation-independent source. The overall transforming activity of luteal-phase PF was verified in an i.p. co-injection xenograft mouse model. Co-injection of Luc-FEXT2 cells with either FF or luteal-phase PF supported early peritoneal implantation, whereas co-injection with follicular-phase PF did not. This study, for the first time, demonstrates that PF from ovulating women can promote different oncogenic phenotypes in FTE cells at different stages of malignant transformation. Most of these activities, other than anoikis resistance and cell migration, are sourced from ovulation.

**Supplementary Information:**

The online version contains supplementary material available at 10.1186/s12935-024-03406-1.

## Introduction

Over the years, the pathological development and etiological mechanism of ovarian high-grade serous carcinoma (HGSC) have become clearer. The earliest recognizable lesion of fallopian tube fimbrial epithelium (FTE) cell transformation involves a *TP53* mutation with an accumulation of mutant p53 protein in a succession of secretory cells, known as the ‘p53 signature’ [1, 2]. Subsequent driver mutations involve amplification of the Rb pathway gene *CCNE1* [3] frequently found in serous tubal intraepithelial carcinoma (STIC), the morphologically fully transformed in situ carcinoma typically found in BRCA1/2 carriers [4] before the development of HGSC in the ovary or peritoneum [5, 6]. Two recent studies employed whole-exome sequencing and mathematical modeling and estimated the mean duration from the inception of an STIC lesion to the onset of ovarian cancer is approximately 6.5 years, with metastases occurring rapidly thereafter [5, 7–10].

The reasons of incessant ovulation as the etiology of HGSC is largely unveiled [11, 12]. Ovulation exposes the fimbriated end of the fallopian tube to the oncogenic contents of follicular fluid (FF), including reactive oxygen species (ROS) [13], IGF2 axis proteins [14], and coagulation cascade and hepatocyte growth factor (HGF) [15]. These oncogenic factors promote the full course of carcinogenesis through mutagenesis, stemness, clonal expansion, and peritoneal seeding [16].

After ovulation, the FF drains into the peritoneal fluid (PF), in which the FTE and transforming lesions are constantly bathed. Additionally, ovarian and intraperitoneal HGSC and exfoliated STIC are directly exposed to PF [17–19]. We aimed to investigate if PF exerts the same spectrum of oncogenic activities as FF, and whether it is sourced from FF. Using cells representing different stages of FTE transformation, we tested the changes in different transformation phenotypes after treatment with FF and PF collected before and after ovulation.

## Materials and methods

### Clinical specimens

This study was conducted under two research programs (TCRD111-042 and MOST 110-2314-B-303-008) and received ethical approval from the Institutional Review Board of the Tzu Chi Medical Center in Taiwan (Approval No. IRB110-238-A).

Follicular fluid was collected during the oocyte retrieval procedure from women who underwent the IVF program in Tzu Chi General Hospital, Taiwan, as previously described [14, 16]. Informed consent was obtained from all the participants. Overall, 27 FF samples were pooled to investigate transformation activities. The PF present in the cul-de-sac was collected from females of reproductive age during laparoscopic surgery in two cohorts: one for the in vitro study and the other for the in vivo IP injection study (Supplementary Tables S1, S2). For the in vitro studies, seven PF samples were collected during the follicular phase of the menstrual cycle, and another seven PF samples collected during the luteal phase were pooled. For the in vivo study, 6 and 14 PF samples were collected at the follicular and luteal phases, respectively, and pooled. As detailed in our previous study [15], PF was aspirated upon entering the peritoneal cavity to reduce contamination from blood or tissue damage. Each sample was centrifuged to obtain the supernatant, which was aliquoted and frozen before pooling and use.

### Cell lines

FTE cell lines simulating different transformation stages of fallopian tube epithelial cells were used [12, 14, 16]. The FT282-V and FT282-CCNE1 cell lines were provided by Dr. Ronny Drapkin [20]. FT282-V is a p53-mutated immortalized cell line representing the non-transformed initiation stage of “p53 signature” [20]. This cell line carries a dominant-negative gain-of-function R175H mutation of the *TP53* gene; FT282-CCNE1 is a subclone of FT282 after transfection with the *CCNE1* gene [20]. This cell line has acquired the ability for AIG (Fig. 3b) but is not fully transformed since it is non-tumorigenic in xenografts. FEXT2 is a fully transformed FTE cell line derived from a p53 and RB disrupted and spontaneously transformed FE25 cells [13] following intraperitoneal xenograft tumorigenesis [16]. These cells were cultured in MCDB105/M199 medium supplemented with 10% fetal bovine serum (FBS) and penicillin/streptomycin (P/S).

### Xenograft tumor model

NOD.CB17-Prkdcscid/JNarl (NOD scid) mice were used for the xenograft tumor-seeding test. In this experiment, 7- to 8-week-old female mice received an intraperitoneal injection of 1 × 10^4^ FEXT2-FLUC cells in a solution of 200 µL of PBS or 10% FF, luteal phase PF, or follicular phase PF. The FEXT2-LUC cell line was derived from a xenograft tumor of FE25 cells transformed by transduction with the luciferase-expressing lentivirus pLAS3w.FLuc.Puro (National RNAi Core Facility of Academia Sinica, Taipei, Taiwan) [16, 21]. The fluid injection was repeated 3 days later and signals in the peritoneal cavity were detected and quantified on the fifth day using an in vivo imaging system (IVIS^®^ by PerkinElmer, Shelton, CT, USA). All mouse-related experimental procedures were approved by the Animal Care and Use Committee of Tzu Chi University (Approval ID: 110 − 35).

### Anchorage independent growth (AIG)

The AIG assay was adapted to a 96-well plate as described previously [16]. In brief, sterilized agarose (Invitrogen, Carlsbad, CA, USA) was aliquoted into 50-mL tubes and subsequently melted and maintained in a 41 °C water bath before use. A bottom layer of 0.8% soft agar in MCDB/M199 medium was placed in each well, and a top layer of 0.4% soft agar mixed with 2000 cells was loaded. Every 3 days, this culture setup was supplemented with 20 µl MCDB/M199 containing 10% FBS was supplemented. After 14 d, the colony numbers were counted in randomly selected fields at 100x magnification.

### Anoikis resistance assay

Anoikis resistance assay was performed using a modified version of the CytoSelectTM 96-Well Anoikis Assay kit (Cat# CBA-081; Cell Biolabs Inc., San Diego, CA, USA) [16]. Briefly, cells were inoculated into 96-well plates coated with agarose at a density of 2 × 10^3^ cells/well and cultured in a serum-free medium. The medium was supplemented with 10% FF, 10% PF, or vehicle, and this was repeated 48 h later. After 3 days, cell viability was determined using the XTT colorimetric assay for 24 h. The results were adjusted by subtracting cell-free background values.

### Ex vivo peritoneal attachment growth assay

For the ex vivo attachment assay, a piece of peritoneum measuring 3 cm × 2 cm was obtained from a female C57BL/6 mouse. After being washed with PBS for 30 min, the peritoneum was placed as an insert in a 48-well chemotaxis chamber (Neuro Probe; Cabin John, MD, USA). The tested cells were labeled with red fluorescent protein (RFP) using TRITC lentivirus (pLAS2w.RFP-C. Ppuro from the Taiwan RNAi core facility) and loaded onto the surface of the peritoneum (2000 RFP-labeled cells) that had been pretreated with 10% FF, 10% PF, or vehicle. The upper and lower chambers were filled with a serum-free culture medium. After 40 min, the surface was washed thrice with serum-free medium and cultured for 24 h in a normal medium containing 10% FBS. The number of RFP-positive cell colonies on the insert was counted using ImageJ software.

### Cell migration and invasion assay

Cell motility was evaluated using a 24-well transwell chamber system (Costar 3422, Corning Inc.). The cells were seeded in the upper chamber at a concentration of 2 × 10^4^ cells in 0.3 mL of serum-free MCDB/M199 media. After 24 h of incubation at 37 °C in a 5% CO_2_ environment, 0.5 mL of medium containing 10% FF was added to both the upper and lower wells. The membranes were then fixed in 4% paraformaldehyde for 20 min, and the migrated cells on the lower surface were stained with Giemsa. For the invasion assay, the transwell membranes were coated with 60 µL of diluted matrix Matrigel (Corning Inc.) overnight. The upper chamber was loaded with 1 × 10^4^ cells. After 48 h, the number of cells that migrated to the lower chamber was counted in three random fields per filter.

### Statistics

Data are presented as mean and standard error. Statistical analyses were performed using GraphPad Prism 8.0 (GraphPad Software, La Jolla, CA, USA) and Microsoft Office Excel 2019 (Microsoft Corp., Redmond, WA, USA). The statistical analysis methods are explained in the figure captions below.

## Results

### FF but not PF modestly promotes the proliferation of FTE cells

**Fig. 1:**
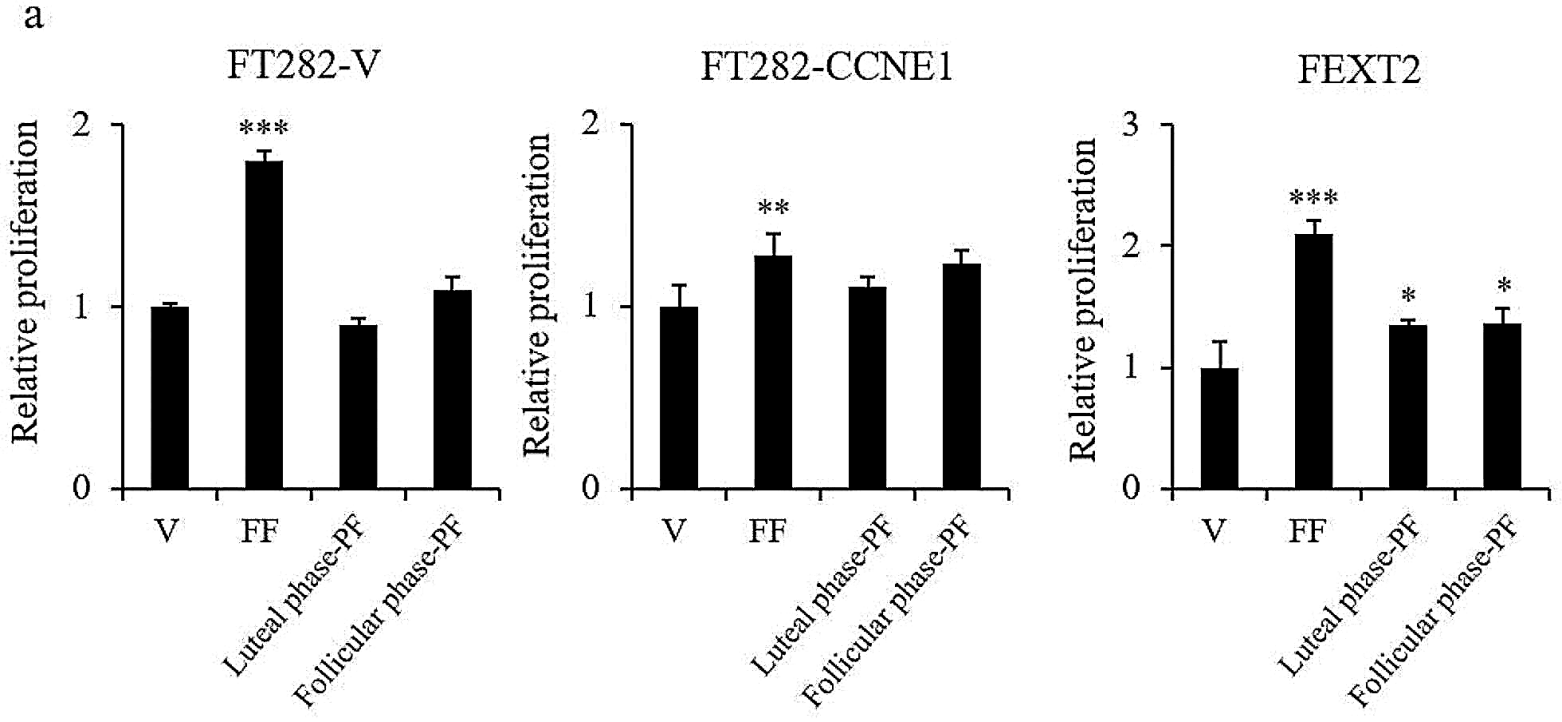
FF but not PF modestly promotes the proliferation of FTE cells. (**a**) Under serum-free conditions, cell proliferation was tested using the XTT assay, and after treatment with vehicle, 10% FF,10% luteal phase PF and 10% follicular phase PF. Cells were harvested after 24 h. Results are from triplicate experiments. Error bar represents mean ± SD (*n* = 4 or more). The asterisk represents a comparison with the vehicle. ** *p* < 0.01, *** *p* < 0.001 by two-sided, unpaired Student’s t-test

Three cell lines representing different states of malignant transformation of FTE were used in this study: FT282-V, FT282-CCNE1, and FEXT2 [16, 20]. Among these, FT282-V mimics the initial “p53 signature” lesion of FTE carrying p53 mutation. In FT282-CCNE1 cells, the CDKN2A/Rb pathway was further disrupted by *CCNE1* overexpression. The cells were partially transformed with anchorage-independent growth (AIG) activity but did not grow tumors in xenografts [16]. FEXT2 is a fully transformed FTE cell line with p53/Rb disruption and AIG and IP tumorigenic capabilities. Figure 1a shows that FF promoted the proliferation of these three cell lines by 1.79-fold, 1.28-fold, and 2.09-fold, respectively. Unlike FF, neither luteal-phase peritoneal fluid nor follicular-phase peritoneal fluid affected cell proliferation. These results suggested that ovulatory FF, but not PF, had a mitogenic effect on FTE cells.

### PF supports anoikis survival and migration of FTE cells independent of the ovulation cycle

**Fig. 2:**
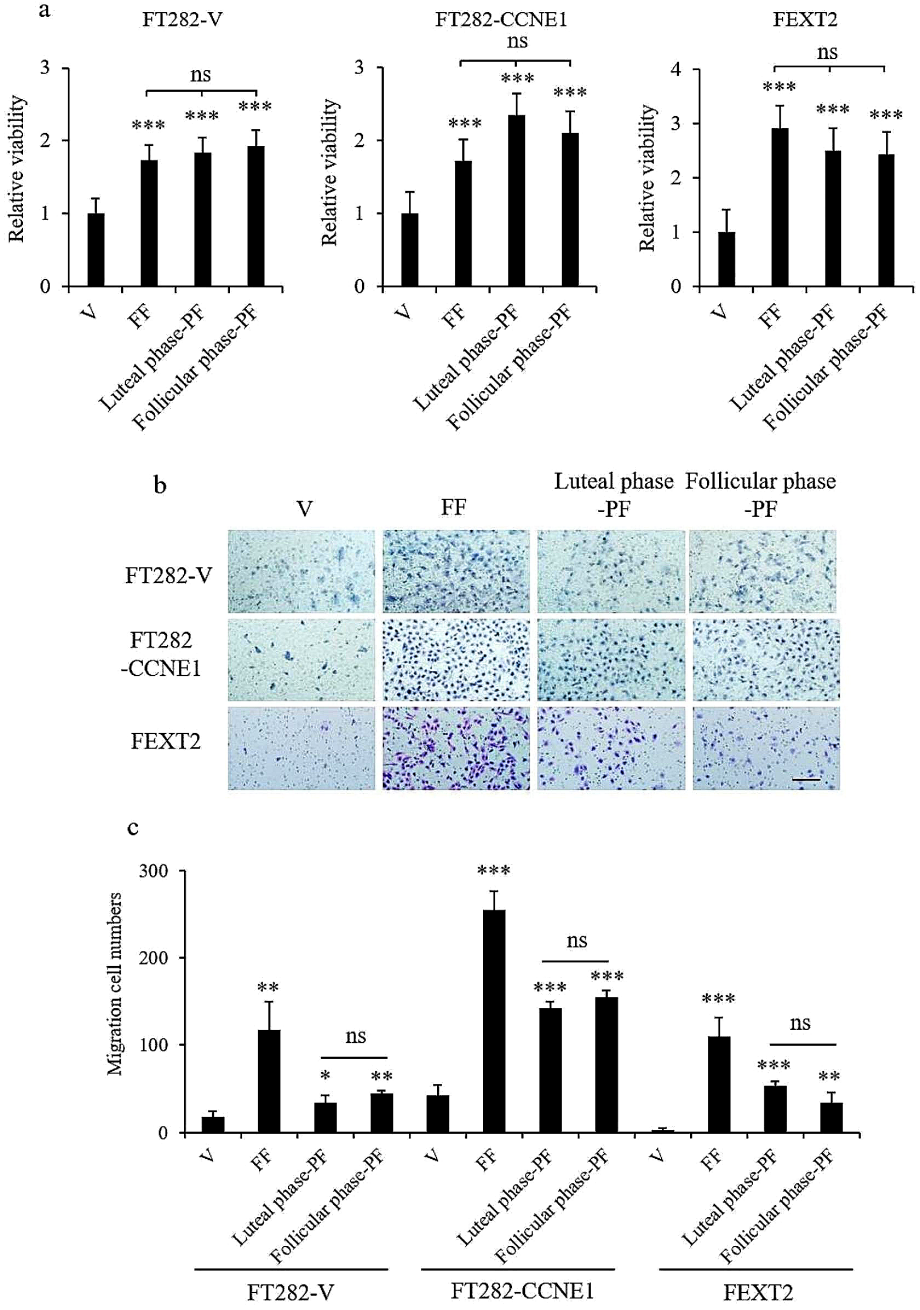
PF supports anoikis survival and migration of FTE cells independent of the ovulation cycle. (**a**) Under the same treatment grouping and condition, 4 × 10^3^ cells were incubated in 0.4% agarose in ultra-low attachment plates. Cell viability was detected by XTT assay after 24 h. (**b, c**) Transwell cell mobility assay was conducted using inserts with 8-µm pores. Representative migration photos (**b**) and comparisons among groups (**c**) are shown. Results were from triplicate experiments. Scale bar: 100 μm. Error bar represents mean ± SD; * *p* < 0.05, ** *p* < 0.01, *** *p* < 0.001

The same panel of tissue fluids and cells was tested for survival in low-attachment cultures. The data showed that both FF and PFs before and after ovulation promoted the survival of detachment-induced anoikis. Viability supported by these three tissue fluids, as compared to vehicle control, was 1.7– 1.9 times higher for FT282-V, 1.7–2.3 times higher for FT282-CCNE1, and 2.4–2.9 times higher for FEXT2 cells (Fig. 2a). There was no difference between FF and PFs in the luteal and follicular phases. Similarly, in the migration analysis, FF, luteal phase PF, and follicular phase PF were found to promote the migration of the three FTES cell lines. FF resulted in 6.43-, 6.02-, and 41.3-fold increase, respectively; luteal phase PF resulted in 1.88-, 3.35-, and 19.9-fold increase, respectively; and follicular phase PF resulted in 2.48-, 3.66- and 12.9-fold increase, respectively, for FT282-V, FT282-CCNE1, and FEXT2 cells (Fig. 2b and C). Notably, while FF generally performed better than PF, there were no differences between luteal phase PF and follicular phase PF in promoting cell migration. The results indicated that PF, similar to FF, supported anoikis resistance and migration of FTE cells, regardless of the menstrual phase.

### Luteal-phase PF promotes AIG of partially and fully transformed FTE cells, likely sourced from FF

**Fig. 3:**
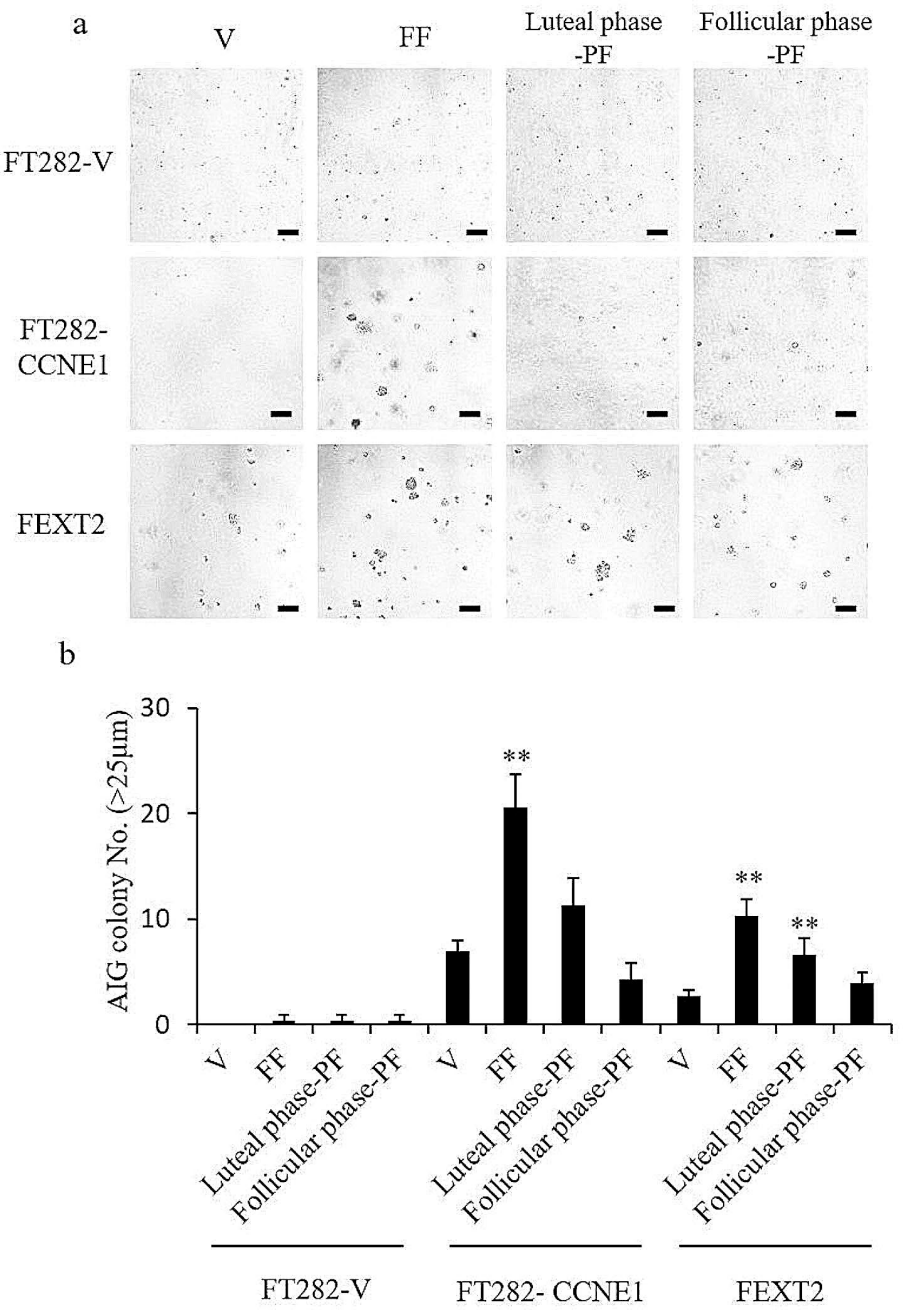
Luteal-phase PF promotes AIG of partially and fully transformed FTE cells, likely sourced from FF. (**a**) Representative photos of the AIG colony. (**b**) AIG of the three cell lines with the same pretreatments. Colonies over 25 μm were counted following 14 days of culture. Results are from three independent experiments. The asterisk represents a comparison with the vehicle group. Scale bar: 100 μm * *p* < 0.05, ** *p* < 0.01, *** *p* < 0.001 by two-sided, unpaired Student’s t-test

As shown in Fig. 3b, FT282-V cells showed no AIG, even after treatment with FF or PF, whereas FT282-CCNE1 and FEXT2 cells showed moderate baseline AIG capacities, which increased by 2.95 and 3.87 folds after FF treatment, respectively. In the presence of luteal phase PF, only FEXT2 showed a significant increase in AIG, whereas FT282-CCNE1 showed no significant increase. In contrast, follicular phase PF did not affect any of the cells (Fig. 3a and b). The results showed that while FF markedly increased the AIG of FTE cells, only luteal phase PF showed a modest promotion effect, likely sourced FF.

### Luteal-phase PF promotes attachment of fully transformed FTE cells whereas FF promotes attachment of all FTE cells

**Fig. 4:**
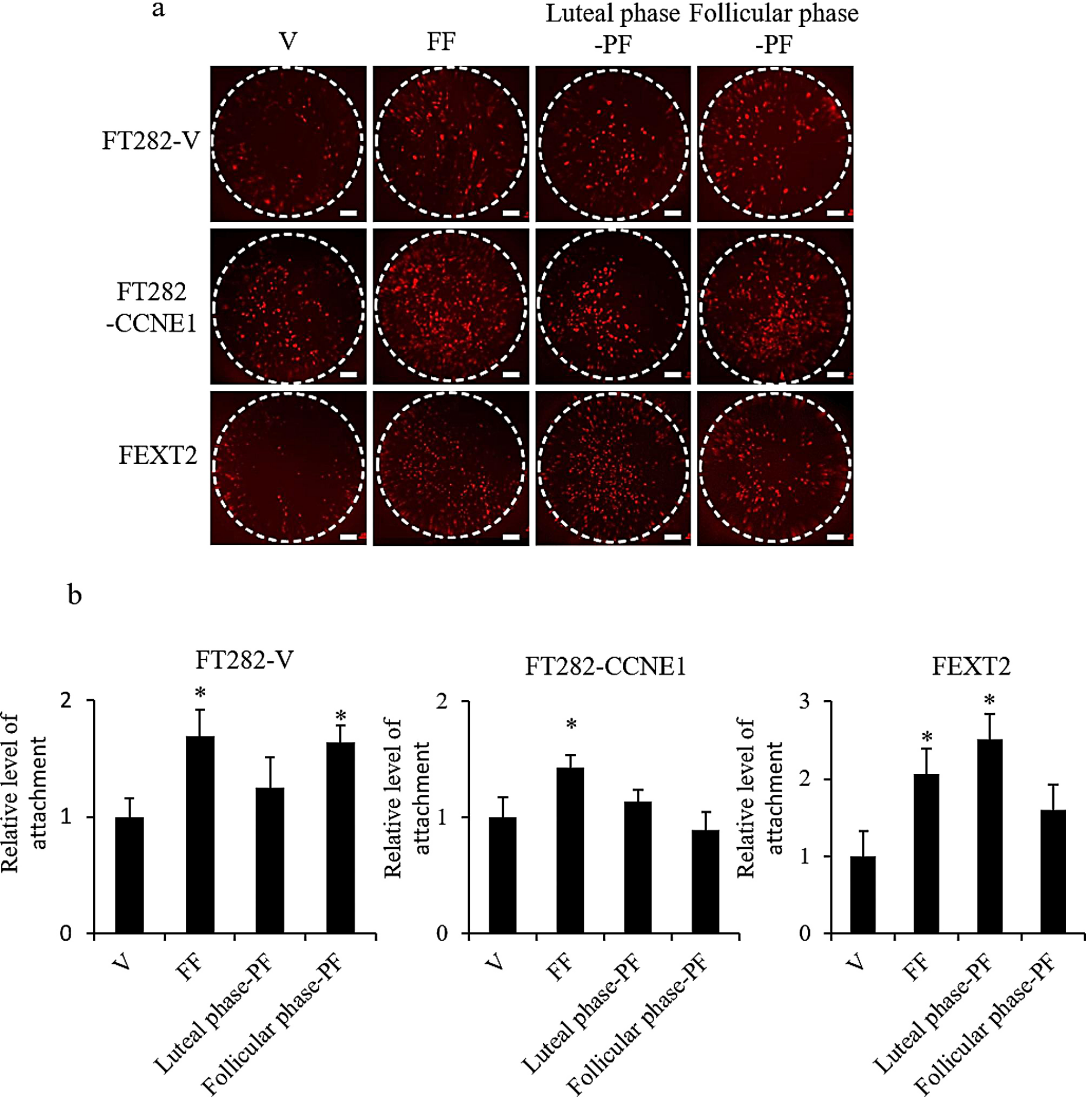
Luteal-phase PF promotes attachment of fully transformed FTE cells whereas FF promotes attachment of all FTE cells. The attachment of RFP-labelled FTE cells to ex vivo-cultured mouse peritoneal tissues after different treatments was examined. The ex vivo culture of the treatment and no-treatment groups of a cell line were performed on the same peritoneum. (**a**) Representative images of cells attached to the peritoneum after 24 h of culture. Scale bar: 200 μm. (**b**) Comparison among the treatment groups. Three or more independent experiments were conducted. The asterisk represents a comparison with the vehicle treatment. * *p* < 0.05, ** *p* < 0.01, by two-sided, unpaired Student’s t-test

We further analyzed the effects of the three fluids on the attachment and growth of FTE cells in the peritoneum cultured ex vivo. As shown in Fig. 4, FF and luteal phase PF enhanced the attachment and growth of FEXT2 cells by 2 folds and 2.51 folds, respectively, but did not have a notable effect on the attachment and growth of FT282-V and FT282-CCNE1 cells. These results indicated that luteal phase PF promoted peritoneal attachment only in fully transformed FTE cells.

### Luteal-phase PF enhances the invasion of less-transformed FTE cells to a lesser extent than FF

**Fig. 5:**
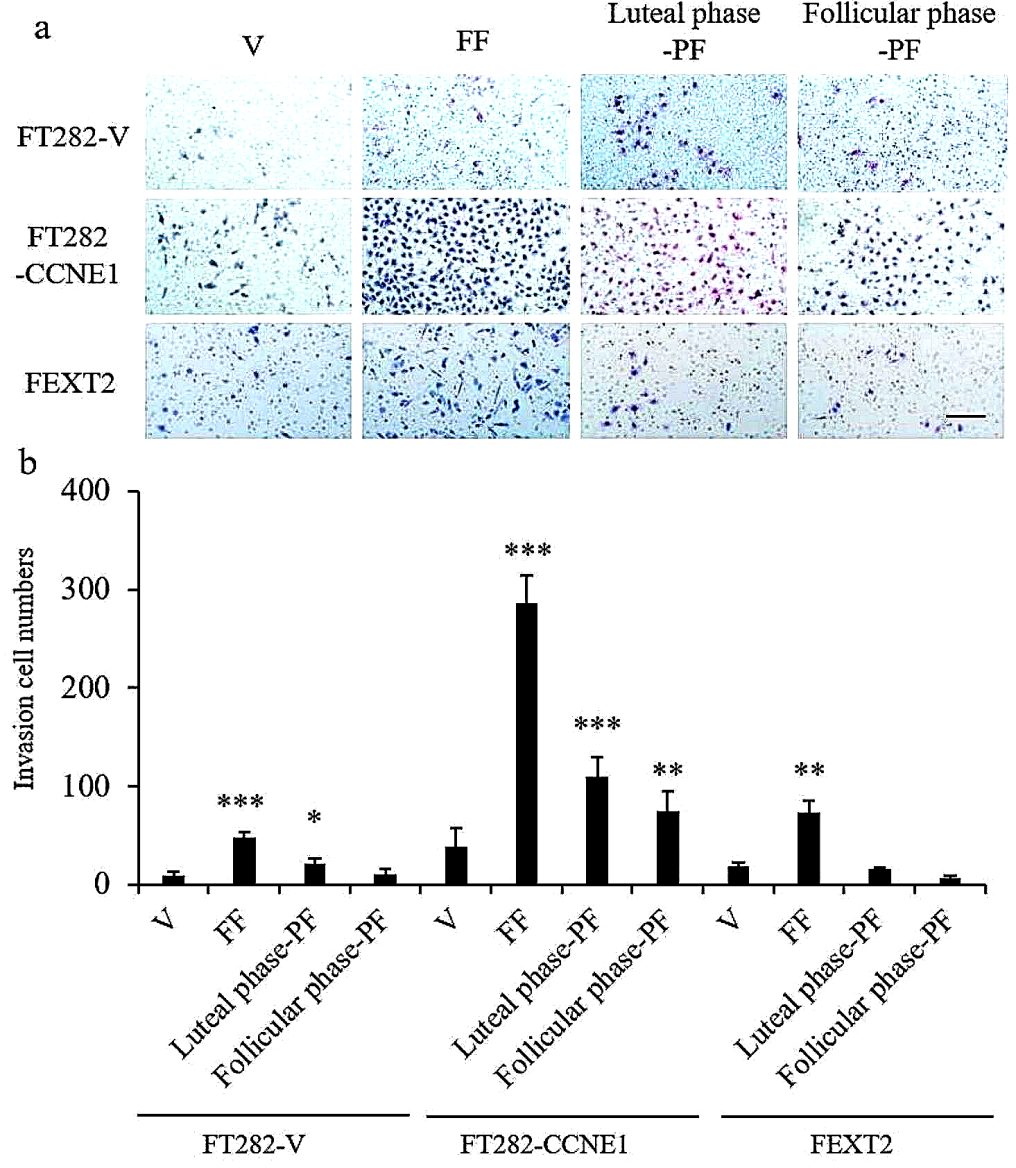
Luteal-phase PF modestly enhances the invasion of less-transformed FTE cells to a lesser extent than FF. Transwell invasion assay was conducted using a matrigel-coated insert with an 8 μm pore size. Growth medium with 10% FF/ luteal phase PF / follicular phase PF or vehicle served as a chemoattractant in two layers of medium, and the number of migrated cells per field was imaged and counted at 48 h. (**a**) Representative photos. Scale bar: 100 μm. (**b**) Comparison of different treatment groups. Data were calculated with mean ± SD from triplicate experiments; * *p* < 0.05, ** *p* < 0.01, *** *p* < 0.001 by two-sided, unpaired Student’s t-test

Matrigel invasion analysis was performed. FT282-V cells showed lower baseline invasion ability than FT282-CCNE1 and FEXT2 cells. FF significantly enhanced the invasion capacity of these cells by 5.03-, 7.53-, and 3.96-fold, respectively. luteal phase PF and, to a lesser extent, follicular phase PF also showed some invasion-promoting activity in the two less-transformed cell lines (FT282-V and FT282-CCNE1), but not in the fully transformed FEXT2 cells. (Fig. 5). These results demonstrated that FF significantly enhances invasion activity. luteal phase PF had a lower promoting effect than FF; however, the effect of luteal phase PF was higher than that of follicular phase PF. This activity in the luteal phase PF is likely derived from ovulation.

### Luteal-phase PF and FF promoted xenograft seeding of transformed FTE cells

**Fig. 6:**
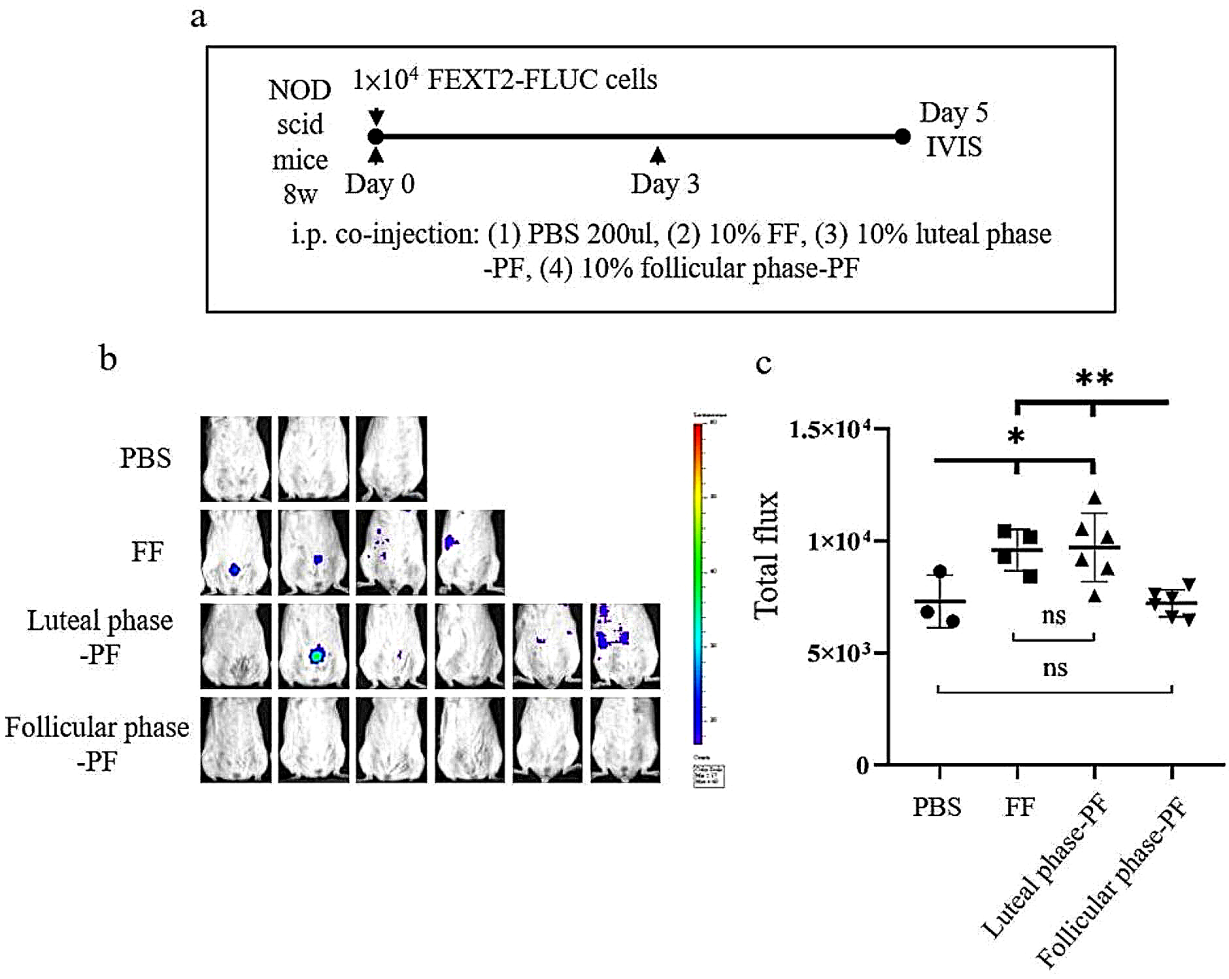
Luteal-phase PF and FF promote early attachment growth of transformed FTE cells. NOD scid mice were intraperitoneally injected with 1 × 10^4^ FEXT2-FLUC cells. (**a**) Cells were co-injected with 10% FF, luteal phase PF, follicular phase PF, or PBS, and the injections were further given twice as boosters. (**b**) Tumor growth from FLUC-labeled FEXT2 cells was detected using an IVIS imaging system. (**c**) The IVIS total radiant efficiency of FEXT2-FLUC intraperitoneal tumors was compared between the groups. The asterisk represents a comparison with the vehicle group, and the hashtag asterisk represents a comparison with the follicular phase PF. * *p* < 0.05, * *p < 0.001 by two-sided, unpaired Student’s t-test

Finally, using a mouse intraperitoneal xenograft model, we tested the effect of FF and PF on the peritoneal seeding of FEXT2 cells in vivo. In this experiment, 10,000 FEXT2-FLUC cells were injected into the peritoneal cavity of NOD scid mice, together with PBS, FF, luteal phase PF, or follicular phase PF. As shown in Fig. 6, on the fifth day after injection, there were significantly more cell signals in the FF- and luteal phase PF -co-injected mice than in the follicular phase PF- and PBS-co-injected mice. These findings indicate that ovulatory FF and PF after ovulation are pro-tumorigenic, whereas PF before ovulation is not.

## Discussion

### Sources of PF in women of reproductive age

Physiologically, PF provides lubrication between the visceral organs and the peritoneum. Its volume positively correlates with the ovulation cycle [22, 23]. Typically, the peritoneal cavity of premenopausal women contains approximately 5–20 ml of fluid [24]. This volume is sourced from the ovaries, mainly ovarian exudates [25, 26]. Ovulation is another source of PF in women of reproductive age. The PF volume is reduced to less than 5 ml in women taking either combined or progesterone-only oral contraceptive pills and is even lesser in postmenopausal women [26]. This confirms that preovulatory ovarian exudate and FF are the major sources of peritoneal fluid.

### Summary of transformation activities of FF and PF before and after ovulation

This is the first study to investigate the effect of PF on the transformation of FTE. In particular, by comparing PF collected before and after ovulation, the role of different sources of PF was clarified. Table 1 summarizes the transformation activities of the FF and the two PFs—luteal phase PF and follicular phase PF. As expected, FF promoted all tested phenotypes in all three FTE cell lines. The magnitudes of the changes were in the following order: cell migration, invasion, AIG, anoikis resistance and peritoneal attachment growth, and in vivo peritoneal seeding. Luteal phase PF seemed to acquire most of these capabilities in cell migration, anoikis resistance, Matrigel invasion, AIG, and peritoneal seeding, affecting a smaller magnitude of change compared with that of FF. However, follicular phase PF exhibited only the migration and anoikis resistance activities. Interestingly, although activities in luteal phase PF and follicular phase PF were generally lower than those in FF, the anoikis resistance activity in both luteal phase PF and follicular phase PF was equal to or even slightly higher than that in FF. Thus, the ovarian exudate source of PF may contribute to the survival of detached FTE cells to the same extent as the FF-sourced one. Indeed, in a study of the ovarian tissue-FTE cell crosstalk using image mass spectrometry, Zink et al. identified norepinephrine secreted from ovarian tissue promoted the migration [27] and, more recently [28], anoikis resistance in the transforming FTE cells.

**Table 1:** Summary of the transformation activities of pre- and post-ovulatory PF on transforming and metastatic FTE cells and possible activity sources

	Basal level (Vehicle)*	10% FF**	Luteal phase 10% PF**	Follicular phase 10% PF**	Luteal VS. follicular phase
Proliferation					
FT282-V	0.12	1.79 ×	NS	NS	Nil
FT282-CCNE1	0.58	1.28 ×	NS	1.23	Nil
FEXT2	0.25	2.09 ×	1.35 ×	1.36 ×	LP = FP
Anoikis resistance					
FT282-V	0.08	1.73 ×	1.84 ×	1.93 ×	LP = FP
FT282-CCNE1	0.16	1.72 ×	2.35 ×	2.11 ×	LP = FP
FEXT2	0.28	2.92 ×	2.51 ×	2.43 ×	LP = FP
Anchorage independent growth (>25 μm)					
FT282-V	0	NS	NS	NS	Nil
FT282-CCNE1	0.67	2.95 ×	1.62 ×	NS	LP > FP
FEXT2	0.33	3.87 ×	2.49 ×	NS	LP > FP
Peritoneal attachment growth					
Ex vivo, FT282-V	49	1.69 ×	NS	1.64 ×	Nil
Ex vivo, FT282-CCNE1	187	1.42 ×	NS	NS	Nil
Ex vivo, FEXT2	124	2.06 ×	2.51 ×	1.60 ×	LP > FP
Migration					
FT282-V	18.17	6.43 ×	1.88 ×	2.48 ×	LP < FP
FT282-CCNE1	42.33	6.02 ×	3.35 ×	3.66 ×	LP < FP
FEXT2	2.66	41.3 ×	19.9 ×	12.9 ×	LP > FP
Matrigel invasion					
FT282-V	9.7	5.03 ×	1.95 ×	NS	Nil
FT282-CCNE1	38.8	7.53 ×	2.83 ×	2.06 ×	LP > FP
FEXT2	18.7	3.96 ×	NS	NS	Nil
Peritoneal implantation in vivo	7.3 × 103 (Flux)	1.31 ×	1.33 ×	0.98 ×	LP > FP

*Scales of transformation phenotypes were: (1) Proliferation: value in XTT assay, (2) Anoikis resistance: value of XTT assay in non- attached culture, (3) AIG: colony (>25 μm) number in soft agar, (4) Attachment growth: fluorescence level of cells attached to mouse peritoneum, (5) Cell migration: number of cells migrated to the lower part of transwell, (6) Matrigel invasion: number of cell in the lower part of matrigel transwell. (7) Peritoneal implantation in vivo: luciferin flux of In Vivo Imaging System

**Folds of change relative to vehicle control were shown. NS: No significant change, LP: lutel phase PF, FP: follicular phase PF

### Transforming activity of PF supports the precursor escape theory of HGSC

This study involved experiments with fully transformed/metastatic FTE cells as well as those in earlier precursor stages. According to the precursor escape theory [29, 30], benign early serous proliferation (ESP) and the later tubal intraepithelial lesion cells may escape from tubal precursor lesions and transform within the peritoneal cavity [17, 29, 31]. A recent study of 50 patients with early-stage EOC revealed the presence of cancer cells in the peritoneal fluid in 12 (24%) patients, and micrometastases in the peritoneum and omentum in another 10 (20%) and 8 (16%) patients, respectively [32]. Studies also showed that ESP and STIL/STIC may have been shed to the peritoneal cavity prior to the formation of overt HGSC [17, 30]. In our animal experiments, xenograft injection using the early passage number of the partially transformed FTE cell line (FE25-P30) showed that the cells were in a non-tumor–forming state, and a small number of cells were seeded in the omentum and survived for more than 5 months (Supplementary Fig. S1). The results of this study provide mechanistic support for this theory, showing that ovulation releases oncogenic activity into the peritoneal cavity to support the transformation of exfoliated cells in the luteal phase and even follicular phase PF of the menstrual cycle.

### Possible transforming factors in PF

Our previous study showed that exposure of ovulatory FF to carcinogens facilitates the full transformation process in the development of HGSC. FF contains various carcinogens, including ROS, proteins of the insulin-like growth factor (IGF) axis, and those of the HGF activation cascade [13, 33]. These carcinogens induce different aspects of malignant transformation of FTE cells including DNA double-strand breaks, stemness activation, clonal expansion, anoikis resistance, AIG, migration, invasion, and xenograft tumorigenesis [14–16, 21]. PF appears to acquire these oncogenic factors and the associated activities from FF. In addition, factors directly involved in peritoneal metastasis may include soluble fibronectin [34] and laminin [35], which are the 4th and 69th most abundant proteins, respectively, in the FF proteome [36]. Additionally, versican secreted by 3D ovarian culture was shown to enhance the migration, invasion, and adhesion of transforming FTE cells [37]; thus, versican is another candidate pro-tumorigenic factor in postovulatory PF.

### Ovulatory and nonovulatory sources of the transforming activity

**Fig. 7:**
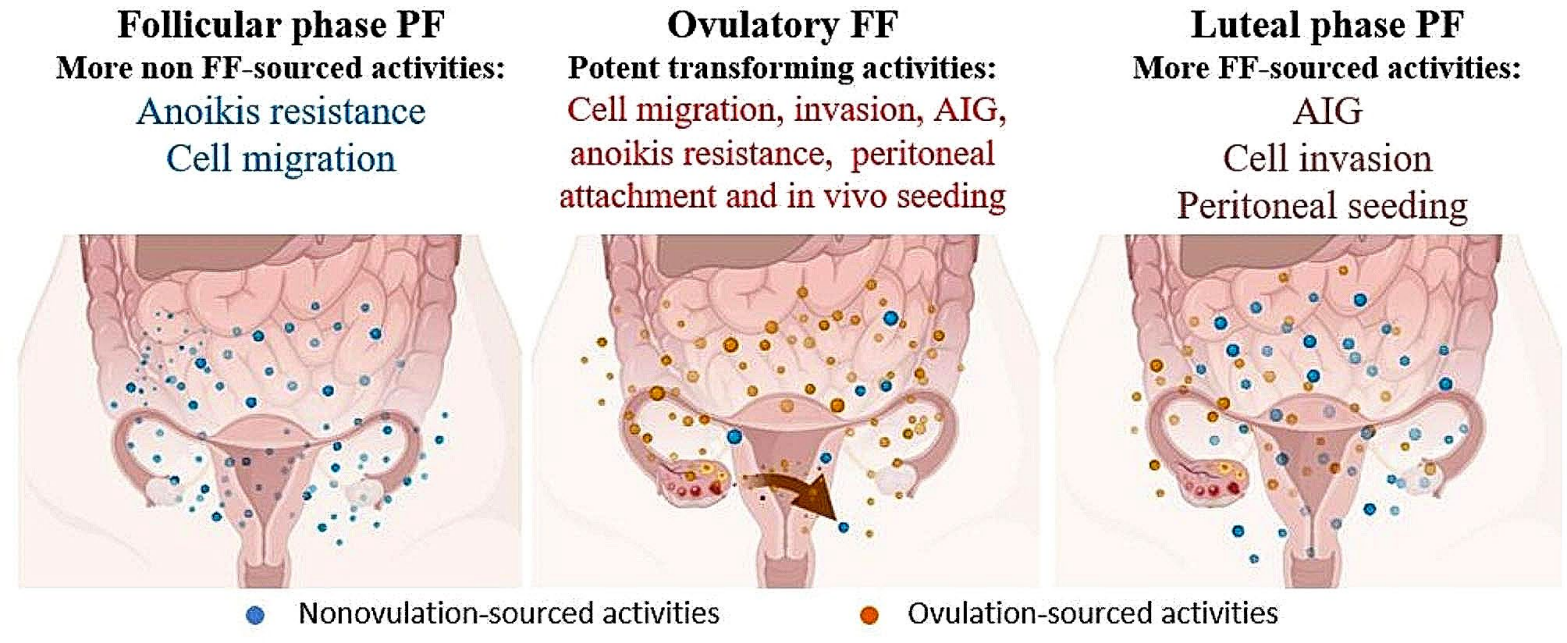
Summary of the transformation activities of FF and PF to different transformation phenotypes of three FTE cellsOvulatory FF is a source of attachment, invasion, and AIG activity, but its effects diminish over time. Conversely, PF was observed at different levels of transformation of FTE-transformed cells, where both anoikis resistance and migration were independent of the menstrual cycle of FF and the phenotype. Additionally, ovulation affects the peritoneal fluid, resulting in invasion and seeding of the peritoneum. Additional phenotypic effects of FF and PF in the three transformed cell lines are outlined above

As depicted in Fig. 7, this study unveiled several oncogenic activities of PF. AIG activity, invasion, and peritoneal seeding were more pronounced in the luteal phase compared to the follicular phase, indicating an association with ovulation. Conversely, anoikis resistance and migration promotion, which exhibited no variation in the PF obtained from either phase, may stem from both ovulatory FF and ovarian exudates. While the nature of the FF transforming activity has largely been disclosed, that of ovarian exudate is less clear but may involve norepinephrine, at the very least.

The results of this study may highlight a strategy of “ovarian rest” in the prevention HGSC, a strategy of putting the ovary not only to an ovulation rest (e.g. using oral contraceptives) but also to an inactive state (e.g. using progestins or gonadotropin-releasing hormone analogs). Based on a plethora of epidemiological studies concluding that oral contraceptive use reduces the incidence of ovarian cancer [38], it was estimated that oral pills have already prevented some 200 thousand ovarian cancers and 100 thousand deaths since its wide use worldwide in the past 50 years [39].

## Electronic supplementary material

Below is the link to the electronic supplementary material.

Supplementary Material 1

## Data Availability

No datasets were generated or analysed during the current study.
