# Correction: Human peritoneal fluid exerts ovulation- and nonovulation-sourced oncogenic activities on transforming fallopian tube epithelial cells

**DOI:** 10.1186/s12935-025-03718-w

**Published:** 2025-03-15

**Authors:** Che-Fang Hsu, Vaishnavi Seenan, Liang-Yuan Wang, Pao-Chu Chen, Dah-Ching Ding, Tang-Yuan Chu

**Affiliations:** 1 Center for Prevention and Therapy of Gynecological Cancers, Department of Medical Research, Hualien Tzu Chi Hospital, Buddhist Tzu Chi Medical Foundation, Hualien, 970 Taiwan; 2 Institute of Medical Sciences, Tzu Chi University, Hualien, 970 Taiwan; 3 Department of Molecular Biology and Human Genetics, Tzu Chi University, Hualien, 970 Taiwan; 4 Department of Obstetrics & Gynecology, Hualien Tzu Chi Hospital, Buddhist Tzu Chi Medical Foundation, 707, Section 3, Chung-Yang Road, Hualien, 970 Taiwan

**Correction: Cancer Cell International (2024) 24:231**

10.1186/s12935-024-03406-1

In this article [1], the committee information under the heading “Xenograft tumor model” and the Ethical approval section was incorrectly given as “Animal Care and Use Committee of Tzu Chi University” but should have been “Animal Care and Use Committee of Tzu Chi Hospital”. The old incorrect and the corrected information are displayed below.

Incorrect text:

“All mouse-related experimental procedures were approved by the Animal Care and Use Committee of Tzu Chi University (Approval ID: 110 − 35).”

Correct text:

“All mouse-related experimental procedures were approved by the Animal Care and Use Committee of Tzu Chi Hospital (Approval ID: 110 − 35).”

## References

[1] Hsu CF, Seenan V, Wang LY, Chen PC, Ding DC, Chu TY. Human peritoneal fluid exerts ovulation-and nonovulation-sourced oncogenic activities on transforming fallopian tube epithelial cells. Cancer Cell Int. 2024;24(1):231. 10.1186/s12935-024-03406-1 PMC11218150 38956560

