# Supplementary material for: Human peritoneal fluid exerts ovulation- and nonovulation-sourced oncogenic activities on transforming fallopian tube epithelial cells
Source: Cancer Cell Int. 2024 Jul 2;24:231. doi: 10.1186/s12935-024-03406-1 (PMC11218150; doi:10.1186/s12935-024-03406-1)

**Supplementary data**


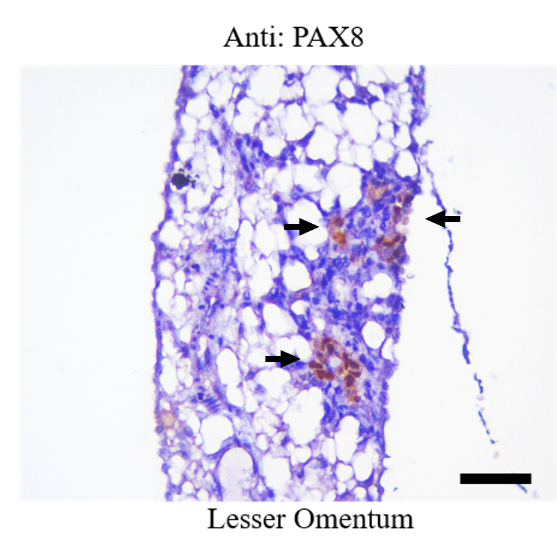


**Supplementary figure 1**: PAX8 IHC identification of long-term seeding of non-transformed human FTE cells (FE25 at passage 30) in the lesser omentum after intraperitoneal injection to NSG mice. The mice were untreated and sacrificed after 5 months. Scale bar, 50 μm.

**Supplementary table 1** Clinical informationof the peritoneal fluid pool for in vitro and ex vivo assay


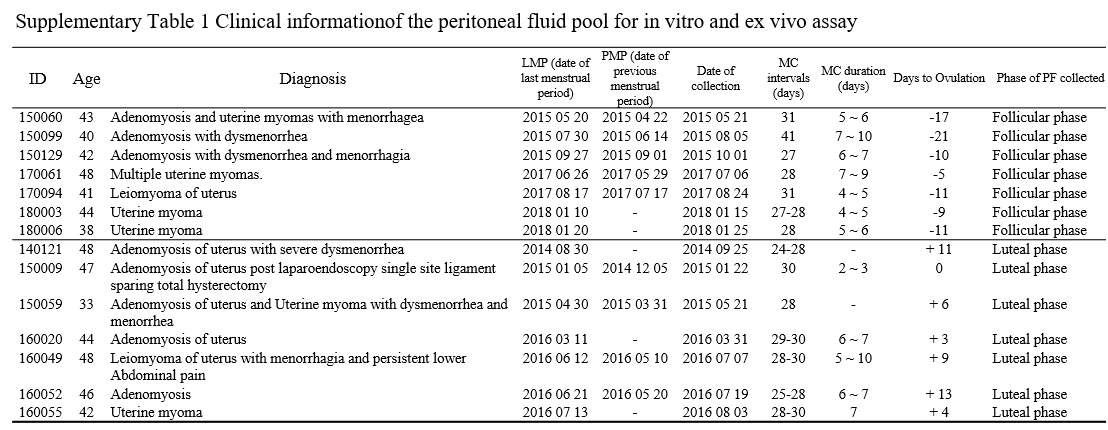


**Supplementary table 2** Clinical information of peritoneal fluid pool for intraperitoneal injection of mice


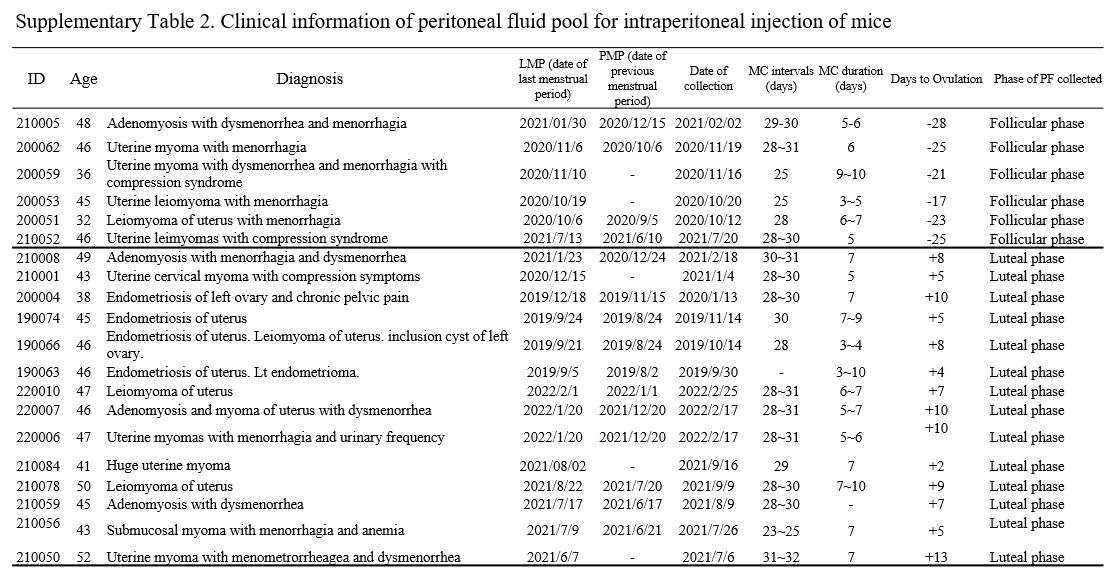

Supplement: Supplementary file 1 — Supplementary Material 1 [file 12935_2024_3406_MOESM1_ESM.docx]
